# Development of Nanofibers with Embedded Liposomes Containing an Immunomodulatory Drug Using Green Electrospinning

**DOI:** 10.3390/pharmaceutics15041245

**Published:** 2023-04-14

**Authors:** Luca Casula, Anže Zidar, Julijana Kristl, Matjaž Jeras, Slavko Kralj, Anna Maria Fadda, Špela Zupančič

**Affiliations:** 1 Unit of Drug Sciences, Department of Life and Environmental Sciences, University of Cagliari, Via Ospedale 72, 09124 Cagliari, Italy; luca.casula@unica.it (L.C.); mfadda@unica.it (A.M.F.); 2 Faculty of Pharmacy, University of Ljubljana, Aškerčeva Cesta 7, 1000 Ljubljana, Slovenia; anze.zidar@ffa.uni-lj.si (A.Z.); julijana.kristl@ffa.uni-lj.si (J.K.); matjaz.jeras@ffa.uni-lj.si (M.J.); slavko.kralj@ijs.si (S.K.); 3 Department for Materials Synthesis, Jožef Stefan Institute, Jamova Cesta 39, 1000 Ljubljana, Slovenia; 4 Nanos SCI, Nanos Scientificae d.o.o., Teslova Ulica 30, 1000 Ljubljana, Slovenia

**Keywords:** simvastatin, liposomes in nanofibers, green electrospinning, immunomodulation, wound dressing, alginate, TEM, MADLS analysis, in vitro safety

## Abstract

Conventional treatments for chronic wounds are often ineffective, thus new therapeutic approaches are needed, such as the delivery of immunomodulatory drugs that can reduce inflammation, restore immune cell function, and facilitate tissue regeneration. A potential drug for such an approach is simvastatin, which has major drawbacks including poor solubility and chemical instability. With the aim of developing a dressing for wound healing, simvastatin and an antioxidant were incorporated into alginate/poly(ethylene oxide) nanofibers by green electrospinning without the use of organic solvents, thanks to their prior encapsulation into liposomes. The composite liposome–nanofiber formulations exhibited fibrillar morphology (160–312 nm) and unprecedentedly high phospholipid and drug content (76%). Transmission electron microscopy revealed dried liposomes as bright ellipsoidal spots homogeneously distributed over the nanofibers. After nanofiber hydration, the liposomes reconstituted in two size populations (~140 and ~435 nm), as revealed by cutting-edge MADLS^®^ analysis. Lastly, in vitro assays demonstrated that composite liposome–nanofiber formulations are superior to liposomal formulations due to a better safety profile in keratinocytes and peripheral blood mononuclear cells. Furthermore, both formulations exhibited similarly advantageous immunomodulatory effects, measured as decreased inflammation in vitro. A synergistic combination of the two nanodelivery systems shows promise for the development of efficient dressings for chronic wound treatment.

## 1. Introduction

Chronic wounds are lesions that cannot be restored in 3 months due to a complex interplay between the immune system, pathogens, and tissue restoration. They represent a major therapeutic challenge, affecting 6.5 million Americans, with an estimated cost of $25 billion per year [1]. These wounds are more often observed in patients with an altered blood supply, a weakened immune system, metabolic disease, medications, or underlying conditions such as diabetes, cancer, malnutrition, or periodontal disease [2]. Frequent attributes are increased cell apoptosis and tissue necrosis, decreased angiogenesis and formation and organization of epithelial tissue, and frequent infections [3].

International guidelines suggest different treatment approaches depending on the location, pathophysiology, and severity of chronic wounds. In addition to treatment of the underlying disease, wound care usually includes wound cleaning and removal of dead tissue, wound compression, and the use of antibiotics [4]. Because these conventional treatment approaches are often ineffective and new insights into the pathophysiology of the wound are available, research is being conducted into new therapies. Particularly, immunomodulatory agents in combination with antibiotics can help reduce inflammation and restore immune cell function [5].

One such promising drug is simvastatin (SIM), as it has both immunomodulatory and antimicrobial activity. Its immunomodulatory effects mainly include decreasing immune cell migration to the wound site and decreasing the production of proinflammatory cytokines and matrix metalloproteinases [6]. The mechanisms underlying its antimicrobial activity remain to be discovered; however, its efficacy has been demonstrated both in vitro and in vivo [7]. Overall, SIM contributes to protecting healthy tissue from degradation and restoring the regenerative process [6,7].

Besides pharmacological treatments, appropriate wound care and dressing is needed to provide a physical barrier, avoid infections, and create a controlled environment that aids and accelerates wound healing [2]. Several nanomaterial-based systems have been introduced into the areas of wound healing and skin regeneration, among which electrospun polymeric nanofibers have been recognized as efficient systems to promote skin regeneration [8]. Owing to their morphological similarity with the extracellular matrix, they provide structural support to the damaged tissues and improve cell growth and proliferation [9,10]. The interconnected fibrous structure ensures gas exchange, nutrient supply, and control of fluid loss, maintaining a moist environment of the wound that augments angiogenesis and collagen synthesis [11].

To facilitate the healing process and improve patient compliance, several active ingredients have been incorporated into nanofibrous dressings, including anti-inflammatory drugs, anesthetics, antimicrobials, and growth factors. Godakanda et al. developed naproxen-loaded ethylcellulose and poly(vinylpyrrolidone) nanofibers to obtain a tunable drug release for the treatment of wound pain and inflammation [12]. Maver et al. prepared a two-layer analgesic wound dressing by electrospinning from the first layer of carboxymethylcellulose and poly(ethylene oxide) (PEO) and using a commercial wound dressing as the second layer. The system was loaded with the non-steroid anti-inflammatory diclofenac and the local anesthetic lidocaine [13]. Similarly, in our previous study, a double layer nanofibrous mat was developed with chitosan/PEO and ciprofloxacin in one layer and poly(ε-caprolactone) with metronidazole in the second layer, resulting in the sustained release of two antimicrobials [14]. In addition, Jin et al. investigated the tissue regeneration activity of gelatin and poly(L-lactic acid)-co-poly(ε-caprolactone) nanofibers loaded with multiple epidermal induction factors, highlighting the potential of the core-shell system as a promising tissue-engineered graft [15].

However, several of these drugs, including SIM [16], have poor aqueous solubility and must thus be dissolved in organic solvents for nanofiber electrospinning. For environmental and health safety and sustainability, water electrospinning (also called green electrospinning) is preferred. For such an approach, poorly water-soluble drugs can first be encapsulated in nanocarriers (e.g., vesicles, micelles, and nanoparticles) to produce nanofibers with incorporated nanocarriers [17]. Liposomes in particular are considered ideal carriers due to their high biocompatibility, low toxicity, biodegradability, and enhanced cellular interaction and uptake [18].

In previous studies, liposomes were successfully embedded into nanofibers by electrospinning polymer solutions with added liposomes. The obtained nanofibers contained up to 10% (*w*/*w*) liposomes [19,20,21]. Another approach presents the preparation of composite polymer/phospholipid nanofibers by electrospinning a solution of polymers and phospholipids in organic solvents, with the aim of obtaining liposomes that can self-assemble after nanofibers hydration [22,23]. However, visualizing the spatial distribution of liposomes/phospholipids in nanofibers and characterizing the self-assembling mechanism after sample hydration or dissolution is a challenging task.

**Figure 1:**
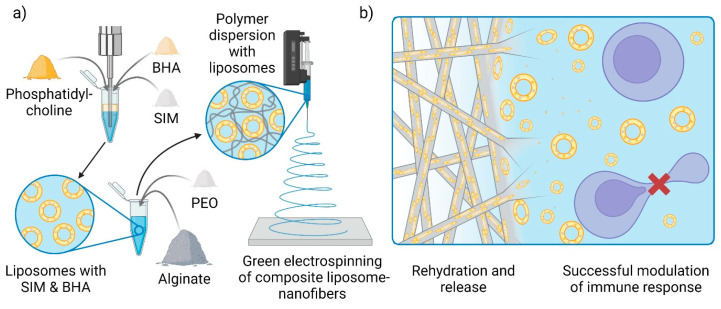
Schematic representation of (**a**) the preparation of the composite liposome nanofibers and (**b**) reconstitution of liposomes after nanofibers hydration and their impact on T-cell receptor activation, consequently inhibiting T-lymphocyte proliferation.

The aim of this work was to develop an innovative nanofiber dressing for wound healing with embedded SIM-loaded liposomes using a green electrospinning technique. For this purpose, SIM was first incorporated into liposomes, and then alginate and PEO at a mass ratio of 80:20 were dissolved in the aqueous liposome dispersion and electrospun (Figure 1a). To prevent degradation and increase the stability of SIM, different concentrations of an antioxidant (butylated hydroxyanisole, BHA) were also added to the liposomal formulations. The nanofibers were characterized in terms of their morphology (with both scanning and transmission electron microscopy), drug loading and release, and chemical stability. Moreover, given the lack of internationally accepted guidelines for the safety assessment of nanofiber delivery systems [24], the safety and efficacy of the developed formulations were individually tested on human keratinocytes and peripheral blood mononuclear cells (PBMC) in vitro (Figure 1b).

## 2. Materials and Methods

### 2.1. Materials

The SIM and BHA were of pharmaceutical grade and supplied by Krka d.d. (Novo Mesto, Slovenia). The phosphatidylcholine (Phospolipon 90G, P90G) was a gift from Phospholipid GmbH (Cologne, Germany). The sodium alginate (MW 138 kDa; Protanal LF 10/60; FMC BioPolymer; Haugesund, Norway) was defined by the manufacturer as 65–75% α-l-guluronate and 25–35% β-d-mannuronate. The following reagents were also purchased: PEO (MW 2 MDa), sodium dihydrogen phosphate monohydrate, and anhydrous disodium hydrogen phosphate (Sigma-Aldrich; Steinheim, Germany); fetal bovine serum (FBS) and Dulbecco’s Modified Eagle Medium (DMEM; Gibco; Paisley, UK); BioTarget^®^ defined serum-free cell culture medium (Biological Industries; Haemek, Israel); Antibiotic Antimycotic Solution (100×; containing penicillin, streptomycin, and Amphotericin B; added at 1% to DMEM and BioTarget^®^; Sigma-Aldrich; Burlington, VT, USA); GlutaMAX™ (added to BioTarget^®^ at 1%; GIBCO Life Technologies; Grand Island, NY, USA); phytohemagglutinin-L (PHA-L; Roche; Basel, Switzerland); dimethyl sulfoxide (DMSO) Hybri-Max^TM^ (Sigma-Aldrich; Gillingham, Dorset, UK); and CellTiter 96^®^ Aqueous One Solution Cell Proliferation Assay consisting of 3-[4,5-dimethylthiazol-2-yl]-5-[3-carboxymethoxy-phenyl]-2-[4-sulfophenyl]-2H-tetrazolium (MTS) reagent (Promega; Madison, WI, USA). The immortalized human keratinocytes (cell line NCTC 2544) were a gift from ICLC, University of Genova, Italy. The PBMCs were isolated from buffy coats of healthy donors, obtained from the Blood Transfusion Center of Slovenia. Water was purified with a Milli-Q system with a 0.22 μm Millipak 40 filter (Millipore, Dublin, Ireland). All the chemicals were used as received, without any further purification or modification.

### 2.2. Liposome Preparation and Characterization

Liposomal formulations were prepared by the direct sonication method. The different components of the formulations were weighed into a glass vial and hydrated with 5 mL of water to obtain liposomes. The dispersions were immediately sonicated (5 s on, 2 s off, 2.20 min three cycles, 40% amplitude) using a high-intensity ultrasonic processor (Cole-Parmer, Vernon Hills, IL, USA). The compositions of the samples are listed in Table 1. Liposomes without SIM and BHA (EMPTY-LIPO) and liposomes without SIM and with 1.2 mg/mL BHA (1.2-BHA LIPO) were prepared by the same procedure and used as controls for the in vitro assays (Section 2.10).

The mean diameter and polydispersity index (PI, a measure of size distribution) of the liposomes were determined by dynamic light scattering (DLS) using a Zetasizer Ultra (Malvern Instrument, Malvern, UK). The samples were backscattered by a helium–neon laser (633 nm) at 174.7° and a constant temperature of 25 °C. The zeta potential was estimated using the Zetasizer Ultra by means of the M3-PALS (mixed mode measurement-phase analysis light scattering) technique, which measures particle electrophoretic mobility.

To evaluate the drug concentrations in the liposomal dispersions and the encapsulation efficiency of SIM in liposomes, the samples were filtered through a syringe filter with pore diameters of 0.45 μm (Sartorius, Göttingen, Germany) to remove undissolved drug crystals. The filtered samples were then ultracentrifuged for 2 h at 100,000× *g* (Ultracentrifuge Sorvall WX100, Thermo Scientific, Waltham, MA, USA) to separate the liposomes from the dissolved unencapsulated drug. All the obtained samples (non-filtrated, filtrated, and centrifuged samples) were diluted and analyzed with ultra performance liquid chromatography (UPLC) to calculate the encapsulation efficiency (*EE*) (Equation (1)):

(1) $$EE\;\left(\%\right)=\frac{SIM\;conc.\;\left(filtrated\;sample\right)-SIM\;conc.\;\left(surpernatant\right)}{SIM\;conc.\;\left(sample\;before\;filtration\right)}\times 100$$

### 2.3. UPLC Analysis

The concentration of SIM was assessed by the UPLC method described by Pohlen et al. [25] using the chromatographic system Acquity UPLC (Waters Corp., Milford, CT, USA) with a UV–VIS photodiode array module equipped with a high-sensitivity flow cell. A reverse phase Acquity UPLC BEH C18 column (1.7 µm; 2.1 × 100 mm; Waters Corp., Milford, CT, USA) was used. A gradient elution containing mobile phase A (water containing 0.1% (*v/v*) formic acid and 10% (*v*/*v*) acetonitrile) and mobile phase B (98% (*v*/*v*) acetonitrile and 2% (*v*/*v*) water) was employed to separate SIM from its degradation product, SIM hydroxy acid, or any form of blank interference. The gradient was achieved by mixing mobile phases A and B at different ratios as follows: starting point 50:50 (A:B); 0–6 min, 50:50–40:60; 6–7 min, 40:60; 7–8 min, 40:60–50:50; 8–10 min, 50:50. The following parameters were used: flow rate, 0.3 mL/min; column temperature, 45 °C; auto-sampler temperature, 10 °C; injection volume, 5 µL; and run time, 10 min. SIM was detected at a wavelength of 238 nm and quantified by summing up the SIM and its active acid form. The calibration curve covered the range 0.5–30.0 μg/mL with R^2^ = 0.9996.

### 2.4. Nanofiber Mat Preparation by Green Electrospinning

All the nanofibers were prepared from solutions with a total polymer concentration of 3.75% (*w*/*w*) by dissolving sodium alginate and PEO (weight ratio 80:20) in water (for empty nanofibers; EMPTY-NF) or by using three liposomal dispersions (0-, 0.6-, and 1.2-BHA SIM LIPO) to obtain the following liposome-loaded nanofibers: SIM LIPO-NF, 0.6-BHA SIM LIPO-NF, and 1.2-BHA SIM LIPO-NF, respectively. The solutions were stirred overnight at room temperature, placed in a plastic syringe, and fixed into the electrospinning machine (Fluidnatek LE100; BioInicia SL, Valencia, Spain). The flow rate of the electrospinning solution was 600 ± 200 µL/h, and the applied voltage was 22 ± 2 kV. For the solutions with BHA, an additional negative voltage of −5 ± 2 kV was applied to the collector. The distance between the needle and the grounded flat collector was 15 cm, and a cycling option in the *y*-axis (100–200 mm with a speed of 8 mm/s) was used to obtain a wider homogeneous nanofiber mat. The entire process was carried out in a climatic chamber with a controlled environment of 37 ± 0.5 °C and 15 ± 2% relative humidity.

### 2.5. Electron Microscopy Analysis

The morphology of the electrospun formulations were evaluated using scanning electron microscopy (SEM) and transmission electron microscopy (TEM). For the SEM, the samples were electrospun in several layers on aluminum foil, which was then properly cut and fixed with double-sided adhesive and conductive tape onto metallic stubs. The samples without sputter coating were observed at different magnifications using high-resolution SEM (235 Supra 35VP-24-13; Carl Zeiss, Jena, Germany) with a secondary detector at an accelerating voltage of 1 kV. The average diameter (d) and its standard deviation (SD) were assessed on the SEM images of at least 50 nanofibers randomly selected using Image J 1.53k software (National Institutes of Health, Bethesda, MD, USA).

To examine the inner structure of the nanofibers without and with liposomes, the samples were electrospun in a thin layer directly onto carbon-coated electron microscopy copper grids (holey, mesh 200; SPI Supplies, West Chester, PA, USA). TEM images of the samples were obtained using the JEM 2100 microscope (Jeol, Akishima, Japan) in the bright field mode and at a working voltage of 200 kV. The vacuum degree was 2.5 × 10^−5^ Pa.

### 2.6. Determination of the Drug Content in the Nanofibers

A sample with an approximate weight of 5 mg (the exact weight was noted) was placed in 5 mL of water and then in an ultrasonic bath to allow complete dissolution of the nanofiber mat. The obtained solution was diluted with methanol and then analyzed with UPLC. Drug loading (*DL*) in the nanofibers was determined according to Equation (2):

(2) $$DL\;\left(\%\right)=\frac{mass\;of\;assessed\;SIM}{total\;mass\;of\;the\;nanofiber\;mat}\times 100$$

The drug loading efficiency (*DLE*) was calculated according to the ratio of the experimental to the theoretical drug content in the nanofibers (Equation (3)):

(3) $$DLE\;\left(\%\right)=\frac{experimental\;DL}{theoretical\;DL}\times 100$$

### 2.7. SIM Stability Study

To evaluate the shelf life of SIM, an accelerated stability test was performed. The prepared nanofiber mats with SIM-loaded liposomes (without and with BHA) were stored in a humidity chamber under elevated stress conditions (40 °C, 75% relative humidity, RH) [26]. After 4 months and 20 days, the drug content of the samples was analyzed to evaluate the chemical stability of SIM, as described in Section 2.6. Degradation in the samples was calculated as the content of SIM relative to the content at the beginning of the stability study.

### 2.8. Release Studies

The SIM release studies were performed under sink conditions using phosphate buffer with sodium dodecyl sulphate (SDS). The phosphate buffer solution was prepared by dissolving 3.39 g of NaH_2_PO_4_·H_2_O and 10.70 g of anhydrous Na_2_HPO_4_ in water and adjusting the pH to 7.4 with a pH meter (SevenCompact, Mettler Toledo, Zürich, Switzerland) and the final volume to 1 L with water. To perform the sink condition experiments, 0.2% (*w*/*v*) SDS was added to the phosphate buffer. To verify the sink conditions, SIM solubility in the buffer solutions was first investigated by adding an excess amount of SIM powder to the prepared buffers. The vials were shaken at 150 rpm and 37 °C, and after 48 h, 2 mL were taken, immediately filtered through a syringe filter with pore diameters of 0.22 μm (Sartorius, Göttingen, Germany), diluted with methanol, and analyzed using the UPLC method.

For the release studies, approximately 10 mg of nanofiber mats were immersed in 15 mL of release medium. The glass vials were shaken at 150 rpm and 37 °C throughout the test. At predetermined time points, 0.5 mL of medium was withdrawn and replenished with fresh medium to maintain a constant volume. The samples were immediately filtered, diluted, and analyzed using UPLC.

### 2.9. Investigation of Liposome Formation after Nanofiber Dissolution

The Zetasizer Ultra (Malvern Instrument, Malvern, UK) was used to investigate liposome formation after dissolution of the nanofibrous samples by the multi-angle dynamic light scattering (MADLS^®^) and particle concentration technique following the manufacturer’s instructions [27,28]. Briefly, approximately 1.22 mg of EMPTY-NF (which corresponds to the same mass of polymers in 5 mg of LIPO-NF) were dissolved in 5 mL of water and analyzed using backscatter analysis to evaluate the dispersant scattering count rate. The LIPO-NF samples (5 mg) were then dissolved in 5 mL water and analyzed with the coupled MADLS^®^-particle concentration technique, setting the dispersant scattering count rate previously obtained from EMPTY-NF as background scattering. The instrument automatically removes the dispersant-background scattering contribution and measures the particle size distribution using MADLS^®^ with detection angles of 12.8, 90.0, and 174.7°.

### 2.10. Evaluation of the Safety and Efficacy of SIM in Nanofibers In Vitro

The in vitro performance of SIM in liposomes and their composite formulations with nanofibers (in both cases without and with BHA), as well as empty liposomes, and empty nanofibers as controls were evaluated. In vitro safety (cell viability) and efficacy (cell proliferation and immunomodulatory activity) were tested using the human NCTC 2544 keratinocyte cell line and PBMCs of healthy donors. Formulations for in vitro testing were prepared under aseptic conditions and then dispersed and diluted in appropriate cell culture medium. Subsequently, 20 µL of each formulation dispersion was added to 80 µL of cell suspension per well in triplicates, using flat or round-bottom 96-well microtiter plates for keratinocytes and PBMCs, respectively. To improve the solubility of hydrophobic SIM, 0.2% (*v*/*v*) cell-grade DMSO was added to the cell culture medium.

#### 2.10.1. Formulation Safety Assay

Safety was evaluated using human keratinocytes and PBMCs of healthy donors by measuring cellular metabolic activity, which also corresponds to cell viability, 72 h after the addition of different formulations. For the keratinocyte viability assay, the cells were seeded at a density of 5 × 10^3^ cells/well in a 96-well flat-bottom tissue culture plate (TPP, Trasadingen, Switzerland) in DMEM medium and incubated overnight in standard conditions (5% CO_2_, 37 °C, 95% humidity) to promote cell attachment. After the initial incubation, the cells were treated for 72 h with formulations with 400, 40, 4, 0.4, and 0 µg/mL SIM. The assay was performed according to the manufacturer’s instructions. The MTS assay is based on the reduction of the MTS reagent to formazan by living cells, which can be detected by measuring the absorbance at 490 nm using a microplate reader (Agilent BioTek Synergy H4, CA, USA). The viability of the treated cells was calculated relative to the untreated cells (controls) as described in Equation (4). The IC50 values were calculated based on a logarithmic regression with a variable slope in GraphPad Prism 8.4.3.

(4) $$Viability\;\left(\%\right)=\frac{Mean\;A_{sample}}{Mean\;A_{control}}\times 100$$

For the PBMC viability assay, the cells were seeded onto a 96-U microplate in BioTarget^®^ defined serum-free medium at a density of 1 × 10^5^ cells/well. Immediately after dispensing the cell suspensions into the wells, two formulation dilutions equivalent to 4 and 40 µg/mL SIM were added to PBMCs in the appropriate wells. The cultures were then incubated for 72 h, and the MTS assay was performed as described above. The controls were cells in the BioTarget^®^ medium without any formulations.

#### 2.10.2. Formulation Efficacy Assay

Two aspects of formulation efficacy were tested, namely tissue repair by keratinocyte proliferation and immunomodulation by lymphocytes in PBMC cultures. To determine the tissue repair potential of the formulations, keratinocytes were cultured in the presence of formulations as described in Section 2.10.1, and cell growth was examined after 24, 48, and 72 h.

The inhibitory effects of formulations on cell proliferation were tested on PBMCs, as they are mainly composed of lymphocytes, among which T-cells can be polyclonally activated with PHA-L, thereby simulating an inflammatory environment present in chronic wounds. The PBMCs were suspended in BioTarget^®^ medium, seeded onto a 96 U-well microplate, and then immediately treated with two different dilutions of formulations containing 4 and 40 µg/mL SIM. The microplates were cultured for 72 h under standard conditions (5% CO_2_, 37 °C, 95% humidity), and then the MTS assay was performed. The reduced proliferation indicated immunomodulatory activity of the tested formulations. The positive controls contained PBMCs with PHA-L, and the negative controls contained only PBMCs. The inhibition of proliferation was calculated as described in Equation (5):

(5) $$Proliferation\;inhibition\;\left[\%\right]=\frac{Mean\;A_{Positive\;control}-Mean\;A_{PHA-L+Formulation}}{Mean\;A_{Positive\;control}-Mean\;A_{Negative\;control}}\times 100$$

### 2.11. Statistical Analysis

All the experiments for the stability and release studies were performed at least in triplicate, and the data are presented as means ± SD. Multiple comparisons of means (one-way ANOVA, post-hoc Tukey HSD test) were used to substantiate the statistical differences between the compared groups, while the Student’s *t*-test was used to compare two samples. The data analysis was performed with the XL Statistic for Microsoft Excel software package. The significance level was set to 0.05.

The release profiles from the nanofiber mats were compared using the statistically derived mathematical parameter known as the similarity factor (*f*_2_):

(6) $$f_{2}=50\times \log\left\{{\left[1+\frac{1}{n}\;\overset{n}{\underset{t=1}{\sum }}{\left(S_{1t}-S_{2t}\right)}^{2}\right]}^{-0.5}\times 100)\right\}$$

where *n* is the number of time points, *S*_1*t*_ is the released percentage of the drug in sample 1, and *S*_2*t*_ is the released percentage of the drug in sample 2, at time *t*. Evaluation of the release profiles was performed using the same time points and was concluded at the first sampling time when drug release was ≥85%. As highlighted by the US Food and Drug Administration and European Medicines Agency, the sameness or equivalence of the two curves is confirmed when the *f*_2_ value is between 50 and 100 [29,30].

GraphPad Prism 8.4.3. was used for the statistical analysis of the in vitro results. To evaluate the statistically significant differences between formulations tested in the PBMC viability and T-lymphocyte proliferation assays, one-way ANOVA was performed in conjunction with Dunnett’s multiple comparisons test. For statistical analysis of the keratinocyte assay, two-way ANOVA was performed in conjunction with Dunnett’s multiple comparisons test. The significance level was set to 0.05.

## 3. Results

### 3.1. Development of Liposomes Containing SIM

To develop the optimal liposomal formulation with high SIM loading and content and small liposome size, different formulations with increasing concentrations of SIM and soy phosphatidylcholine were prepared in a preliminary study (Table S1). The maximum achievable concentration of SIM is 20 mg/mL, as the tested formulations with 25 and 30 mg/mL SIM resulted in the formation of aggregates and/or precipitated components and were, therefore, not suitable for further studies. Liposomes with 100 mg/mL P90G and 20 mg/mL SIM were demonstrated to be the most promising, with an average diameter of 65 nm, zeta potential of −14 mV, and very high encapsulation efficiency of 96% (Table 1). As the concentration of SIM in the dispersion was 19 ± 1 mg/mL, which is near the theoretical one (20 mg/mL), no or minimal drug degradation occurred during the sonication process. To prevent oxidative degradation of SIM, BHA, a synthetic antioxidant commonly added to drugs, food, cosmetics, and other products to prevent oxidative degradation [26,31], was added to the formulations. Increasing the BHA concentration increased the average diameter of the obtained liposomes, ranging from 65 nm (0 mg/mL BHA) to 106 nm (1.2 mg/mL BHA). Nevertheless, even the formulation with the highest BHA concentration exhibited a narrow size distribution of the liposomes. All the formulations showed a negative zeta potential and high encapsulation efficiency, which decreased with increasing BHA concentrations (Table 1).

### 3.2. Development of Composite Liposome Nanofibers

**Figure 2:**
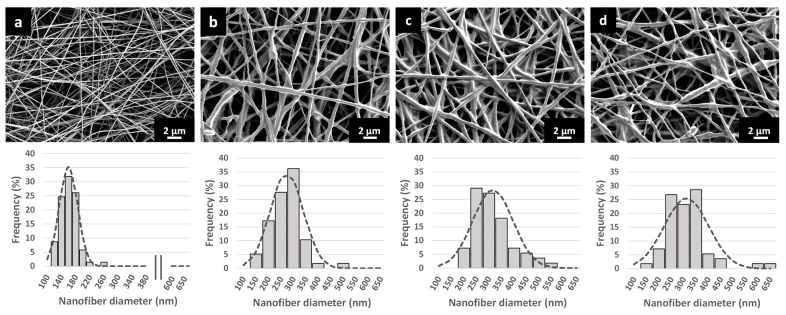
SEM images (upper panel) and diameter distribution (lower panel) of (**a**) EMPTY-NF, (**b**) SIM LIPO-NF, (**c**) 0.6-BHA SIM LIPO-NF, and (**d**) 1.2-BHA SIM LIPO-NF.

In this study, the biopolymer alginate was chosen due to its biocompatibility and non-toxicity. Dry alginate dressings can absorb wound fluids and form a gel-like system that can maintain a physiologically moist environment, reduce bacterial contaminations, and facilitate new tissue formation and rapid re-epithelialization [32,33]. However, alginate electrospinning is challenging due to its polyelectrolyte nature and chain conformation characteristics, thus a blend polymer solution of alginate and PEO was used [34]. To obtain the optimal process and formulation parameters for preparation of nanofibers with high alginate content, a preliminary study was performed without liposomes. Of all the tested polymer concentrations (3, 3.25, 3.5, 3.75, and 4% (*w*/*w*) with a constant alginate/PEO ratio of 80:20), 3.75% was the most suitable polymer concentration according to the spinnability and morphology of the nanofibers (Figure 2a).

Considering SIM is poorly soluble in water, and to avoid using organic solvents, green electrospinning was achieved by adding polymers to liposomal dispersions. Green electrospinning is employed for fabricating nanofiber mats for tissue engineering and regenerative medicine and reduces the drawbacks of using organic solvents, such as issues regarding environmental safety and human toxicity due to residual solvent impurities [35]. As all the liposomal formulations exhibited promising characteristics, three of them were selected for the preparation of the polymeric dispersions for liposome loading and electrospinning: 0-, 0.6-, and 1.2-BHA SIM LIPO. LIPO-NF electrospinning was less stable than EMPTY-NF. Additionally, the antioxidant-containing samples required an additional negative voltage on the collector to obtain an optimal Taylor cone and maintain a stable process. The nanofibers were then characterized in terms of morphology, drug loading and release, and chemical stability of the drug.

### 3.3. Characterization of the Nanofibrous Scaffolds

The nanofibers were theoretically composed of 62.9% P90G, 24.5% polymers, and 12.6% SIM, presenting a very high content of both phospholipids and drug. The extent of the experimental drug loading was slightly lower than the theoretical one, and all three formulations reached a drug loading efficiency of approximately 80% (Table 2). In particular, the BHA-loaded samples showed slightly lower average values than those of SIM LIPO-NF, which, however, were not statistically significant.

The SEM images of EMPTY-NF (Figure 2a) show smooth, beadless nanofibers with homogeneous surfaces and an average diameter of approximately 160 nm. By contrast, when liposomal dispersions were used (instead of water) as a vehicle to prepare polymeric solutions, the composed LIPO-NF (Figure 2b–d) were thicker, had rougher surfaces, contained sphere-like structures, and had merged nanofiber cross-sections. This is probably due to the higher content of phospholipids and drugs in the nanofibers, which was 76% in total. Similarly, Cui et al. prepared chitosan nanofibers with liposomes and observed rough surfaces with small clusters associated with the presence of immobilized liposomes [36]. In our study, the average nanofiber diameter of SIM LIPO-NF was ~270 nm, and the addition of BHA further increased the diameter to more than 300 nm (Table 2).

**Figure 3:**
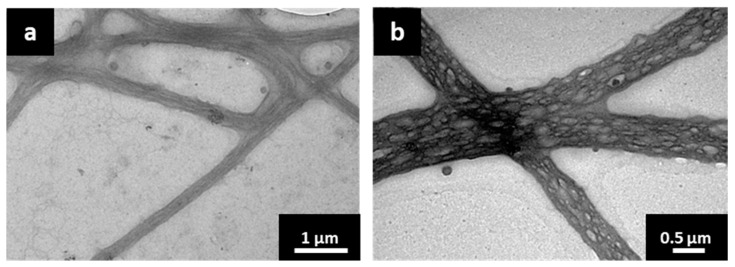
TEM images of (**a**) EMPTY-NF with fibrous inner structure and (**b**) SIM LIPO-NF with brighter compartmental spots, which might be due to evaporated water from the liposomes.

The visualisation of liposomes or phospholipid structures in nanofibers is important since the electrospinning process might change the phospholipids’ spatial arrangement with a consequential loss of the liposomal structure. For the investigation of the embedded liposomes in nanofibers, most studies reported SEM images where the nanofiber surface and shape can be observed [36,37,38]. By contrast, to the best of our knowledge, TEM has never been used for the evaluation of incorporated liposomes in monolithic nanofibers, but only for core-shell types [39,40,41]. In our study, the nanofibers interacted with the hydrophobic carbon foil after direct deposition onto the grid, which resulted in an increased nanofiber diameter. The TEM images of EMPTY-NF revealed a fibrous structure with a linear bundle orientation of polymer chains in the nanofibers (Figure 3a). The inner structure of SIM LIPO-NF differed from EMPTY-NF because it contained many compartmental structures with a high density of electronically less dense ellipsoidal spots, which might represent empty spaces remaining after water evaporation from the liposomes (Figure 3b). Inside the core-shell nanofibers, Li et al. also observed bright elliptic shapes in the core, described as liposomes that obtained such morphology due to the stretch along with the jet fluid during the electrospinning process [41].

### 3.4. In Situ Liposomes Formation after Nanofiber Dissolution

The TEM micrographs revealed dried liposomal structures in the nanofibers. Therefore, the interaction with water after hydration and dissolution of the composite systems might lead to reconstitution of the SIM liposomes (Figure 1b). Using DLS, Laidmäe et al. analyzed the particle sizes of self-assembled liposomes obtained after hydration of polyvinylpyrrolidone/phosphatidylcholine nanofibers. To prevent anomalous results, the authors ultracentrifuged the samples and removed the supernatant containing the polymer [42]. By contrast, in our current study, the morphology of the reconstituted liposomes was explored by removing any possible scattering interferences of the polymers using the innovative particle concentration technique coupled with MADLS^®^. This high-resolution technique combines scattering information from multiple angles (backscatter, side scatter, and forward scatter), providing better insight into the particle size distribution of the sample. The particle concentration technique can be considered an extension of MADLS^®^ that can provide the total particle concentration and particle concentration of each size population by eliminating dispersant scattering [27,43]. As such, it has become remarkably important in the characterization of micro- and nano-sized systems [44,45,46,47].

Prior to the experiments, EMPTY-NF were dissolved in water and analyzed to obtain the background dispersant scattering value. The LIPO-NF samples were then also dissolved and analyzed. Two main size populations were formed (Table 3). One was characterized by an average diameter that varied between 135 and 154 nm, whereas the other was ~430 nm. The relative intensity of the two peaks slightly varied, showing no or low predominance of one population over the other. These results indicate that two populations of liposomes form after nanofiber dissolution. The formation of rehydrated liposomes is important because such liposomes can affect drug efficiency and toxicity by increasing drug uptake into cells.

### 3.5. SIM Release from Nanofibers

**Figure 4:**
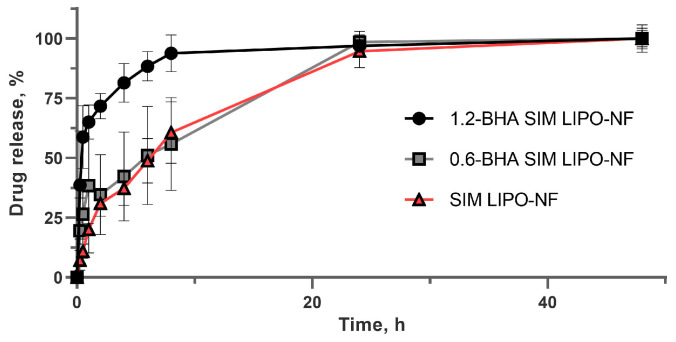
SIM release profiles from the nanofibers with embedded liposomes under sink conditions expressed as % of SIM released over time. The data represent the average values ± SD (*n* = 3).

SIM release from the nanofibrous samples was evaluated under sink conditions. The European Pharmacopoeia defines sink conditions as a volume of release medium that is at least 3–10-fold greater than the saturation volume [48]. The SIM solubility was 22.6 ± 1.7 µg/mL in a phosphate buffer (pH 7.4) and 717.7 ± 11.5 µg/mL in a phosphate buffer (pH 7.4) with 0.2% SDS (*w*/*v*) (sink release medium). The highest theoretical SIM concentration achievable in release medium is 70 µg/mL; therefore, sink conditions were achieved. The release profiles of the three formulations are shown in Figure 4. SIM LIPO-NF and 0.6-BHA SIM LIPO-NF showed similar release profiles under sink conditions, as confirmed by the calculated similarity factor (*f*_2_) (Table 4), releasing approximately 50% of SIM after 5–6 h and 100% of SIM after 24 h. Conversely, 1.2-BHA SIM LIPO-NF exhibited a faster release, releasing almost 70% and 100% of SIM after 1 h and 10 h, respectively. Since the same conditions and spinning time were used to prepare the formulations, resulting in the same size and thickness of the samples, we hypothesize that the different release profile could be attributed to the irregular nanofiber morphology of 1.2- BHA SIM LIPO-NF. Given the complexity of the system studied, the drug could diffuse from phospholipids still embedded in the nanofibers, and/or liposomes could first be released from the nanofibers and then release the drug.

### 3.6. Chemical Stability of SIM in the Nanofibers

**Figure 5:**
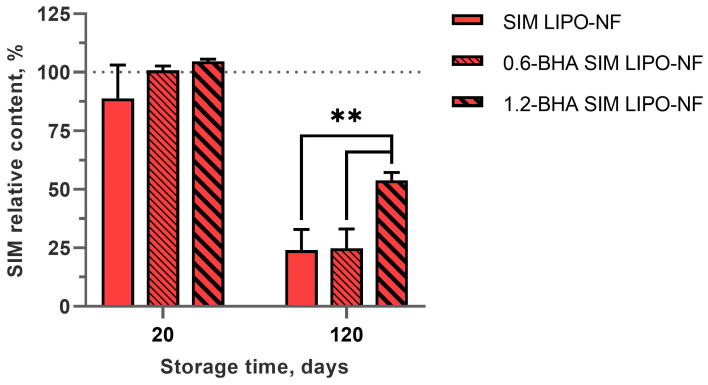
SIM relative content for the three nanofibrous formulations expressed as percentage of SIM relative to the initial drug content after 20 days and 4 months at accelerated storage conditions (40 °C; 70% RH). The data represent the average values ± SD (*n* = 3). ** (*p* < 0.01).

SIM is an unstable molecule due to its oxidative reactivity, thus antioxidants have often been used to prevent or reduce its degradation [26,49]. For this purpose, the chemical stability of SIM in different nanofibrous formulations with increasing BHA concentrations was evaluated with accelerated stability conditions (40 °C; 70% RH). BHA prevented drug degradation after 20 days of storage, whereas some degradation occurred in the samples without BHA. Nevertheless, the variability among the replicates was large, thus the antioxidant-loaded samples did not exhibit significant differences after 20 days (Figure 5). Conversely, the SIM content of the samples stored for 4 months was decreased. Particularly, the remaining drug content in SIM LIPO-NF and 0.6-BHA SIM LIPO-NF was 24% and 25%, respectively. The sample loaded with more antioxidant (1.2-BHA SIM LIPO-NF) exhibited reduced drug degradation after 120 days, with the SIM content more than two-fold higher (54%) than that of the other samples (Figure 5). Sterle Zorec et al. observed 96% retention of SIM content after 1 month of storage at accelerated conditions in dry polyvinylpyrrolidone/BHA particles, whereas particles without the antioxidant retained only 6% of SIM [26]. They showed complete pristine SIM degradation after 2 months at room temperature and low humidity, and thus the proposed composite system in our current study might better protect SIM from degradation.

### 3.7. In Vitro Safety and Efficacy Assessment of SIM Formulations

The safety of the formulations was screened by in vitro cell viability tests using human immortalized keratinocytes and PBMCs. The efficacy of the formulations was determined by proliferation assays on keratinocytes and PHA-L-stimulated T lymphocytes. The aim of these experiments was to assess whether the SIM composite liposome–nanofiber formulations increase keratinocyte proliferation (which would suggest faster epithelial tissue repair) or inhibit PHA-L-stimulated T-lymphocyte proliferation (which would suggest anti-inflammatory activity).

#### 3.7.1. In Vitro Safety Profiles of SIM Formulations

**Figure 6:**
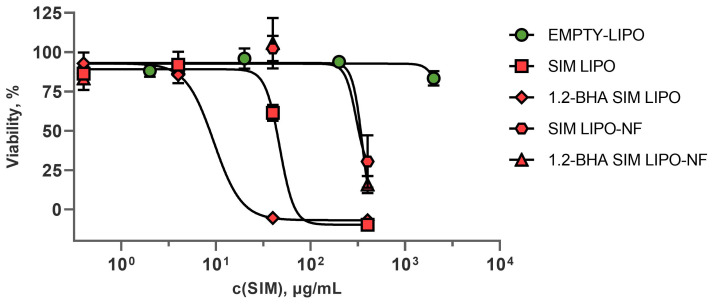
The effects of the formulations with different SIM concentrations on keratinocyte viability. Formulations without and with SIM are indicated in red and green, respectively. The data are presented as means ± SD (*n* = 3).

Treatments with different dilutions of the formulations revealed that <4 µg/mL SIM does not affect keratinocyte viability (Figure 6). To compare the safety of the formulations, the half-maximal inhibitory concentrations (IC50 values) required to inhibit growth or kill 50% of keratinocytes were calculated for each formulation. The SIM-loaded liposomes had lower IC50 values than those of the liposome–nanofiber formulations, indicating that incorporating SIM-loaded liposomes into nanofibers improves the safety profile of the formulation (Table 5). This is most probably due to the slower release of SIM-containing liposomes from nanofibers and/or the different size distribution of liposomes after release from nanofibers. Sadeghi-Aliabadi et al. obtained similar results, i.e., the IC50 value of pure SIM for HeLa cells was 9 µM or 3.8 µg/mL [50]. Additionally, BHA influenced the safety of the liposomal formulations, as BHA-loaded liposomes showed decreased safety and IC50 values by five-fold. Conversely, this effect was not detected in the equivalent formulations with liposomes and BHA incorporated into nanofibers (Table 5).

**Figure 7:**
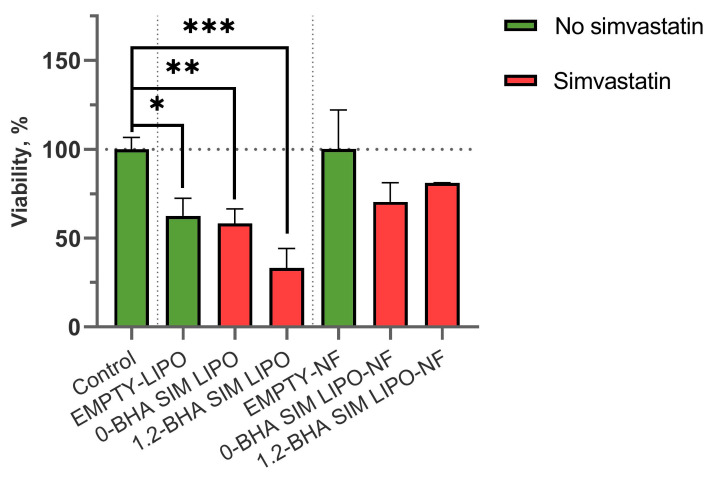
The effects of the formulations containing 4 μg/mL SIM on the viability of PBMCs. The data are presented as means ± SD (*n* = 3). * *p <* 0.05, ** *p <* 0.01, *** *p <* 0.001.

Two concentrations of SIM tested on keratinocytes were selected for the assay with PBMCs. The concentration that proved to be safe in all formulations was 4 μg/mL of SIM, whereas 40 μg/mL of SIM was safe in nanofiber formulations but not in liposomal ones. The PBMCs were more sensitive to SIM formulations, as all formulations containing 40 μg/mL SIM were highly toxic. However, formulations containing 4 μg/mL SIM showed similar results as those in the keratinocyte assay. Compared to the untreated cells, the liposomal formulations decreased PBMC viability, whereas composite liposome–nanofiber formulations did not. Furthermore, the addition of BHA to liposomal formulations decreased PBMC viability. However, these effects were not detected in composite liposome–nanofibers with BHA, indicating that liposome release rates may play an important role (Figure 7).

Interestingly, the metabolic activity of PBMCs in the presence of EMPTY-NF, i.e., polymeric nanofibers only, was the same as that in the control sample containing only medium without any formulation. Conversely, EMPTY-LIPO significantly (*p* < 0.05) decreased the metabolic activity of PBMCs (Figure 7). Our findings regarding the safety of using liposomes in keratinocytes and PBMCs are in agreement with the scientific literature. Namely, the bronchial epithelial cell line showed a good safety profile of liposomes used in our tested concentration range [51]. However, PBMCs and lymphocytes were found to be increasingly susceptible to phospholipids already at lower concentrations equivalent to those in our formulations that contained 4 μg/mL SIM [52].

#### 3.7.2. In Vitro Efficacy of SIM Formulations

**Figure 8:**
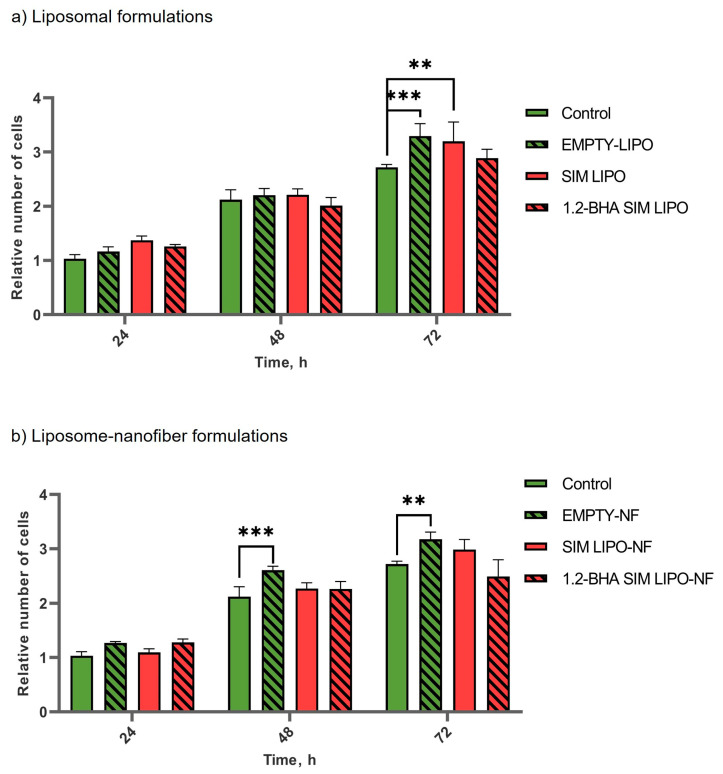
The effects of (**a**) liposomal formulations and (**b**) composite liposomal-nanofiber formulations on keratinocyte proliferation. Data are presented as means ± SD (*n* = 3). ** *p* < 0.01, *** *p <* 0.001.

The tissue regeneration potential of the newly developed nanodelivery systems was determined by measuring keratinocyte proliferation after treatment with various formulations containing 4 µg/mL SIM, assessed after 24, 48, and 72 h. No significant differences in keratinocyte proliferation were detected for SIM formulations, except for liposomes without and with SIM that exhibited increased proliferation at 72 h; however, this result lacks consistency at the other time points, thus no conclusions can be made. Conversely, empty nanofiber formulation (EMPTY-NF) showed significantly increased proliferation after 48 and 72 h. Even though the nanofibers were dispersed in the cell medium beforehand and only small fragments remained, it seems that this was enough to induce cell growth (Figure 8).

**Figure 9:**
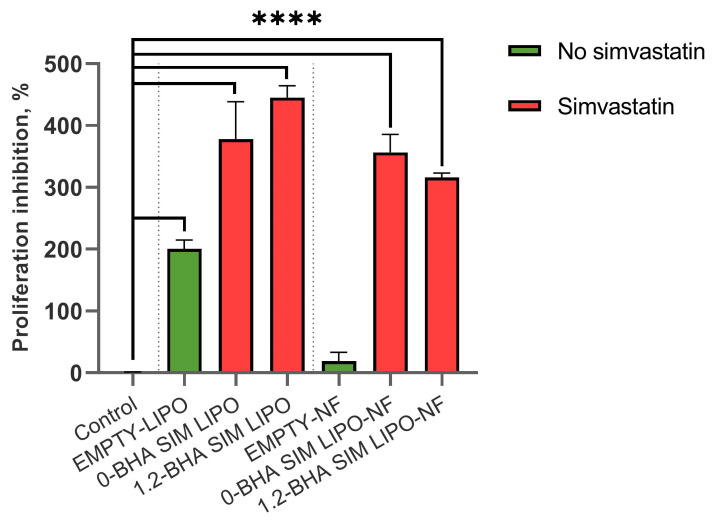
The inhibitory effects of the formulations on PHA-L-induced T-lymphocyte proliferation. Data are presented as means ± SD (*n* = 3). **** *p* < 0.0001.

To determine the immunomodulatory effects of the formulations, the PHA-L-induced T-lymphocyte proliferation assay was used, as this model simulates inflamed conditions that are present in chronic wounds. The formulations containing 4 µg/mL SIM were considered non-toxic for keratinocytes (Figure 6) and were thus used in these experiments. All tested formulations with SIM significantly inhibited induced T-lymphocyte proliferation (Figure 9), which could contribute to alleviating inflammation and in turn decrease local tissue destruction and promote its regeneration [53]. Furthermore, EMPTY-LIPO formulations themselves also inhibited induced T-lymphocyte proliferation by 200%, whereas the addition of SIM to liposomes increased the inhibition by approximately two-fold (SIM LIPO and 1.2-BHA SIM LIPO by 378% and 445%, respectively). Conversely, EMPTY-NF did not significantly inhibit PHA-L-induced T lymphocytes, whereas composite liposome–nanofiber formulations showed similar effects as liposomal formulations (SIM LIPO-NF and 1.2-BHA SIM LIPO-NF increased inhibition by 356% and 315%, respectively). These effects can be observed because SIM impacts T-lymphocyte activation by blocking their T-cell receptor signaling activation pathway. This pathway is activated in T lymphocytes after their recognition of antigens presented within MHC molecules in vivo and after their polyclonal activation by PHA-L in vitro [54,55].

These results confirm the superiority of the composite liposome–nanofiber formulations compared to only liposomal formulations due to their better safety profile on both keratinocytes and PBMCs (Figure 6 and Figure 7). Furthermore, both formulation types exhibit a similar efficacy in reducing PHA-L-stimulated T-lymphocyte proliferation (Figure 9), thereby diminishing the extent of inflammation.

## 4. Conclusions

Poorly water-soluble SIM was successfully incorporated into alginate/PEO nanofibers by electrospinning polymer dispersions with high concentrations of SIM-loaded liposomes. We chose to avoid organic solvents and use so-called green electrospinning as well as alginate, a renewable and biodegradable biopolymer, to contribute to sustainable development, which enables more efficient use of resources and creates less stress on humanity and the environment. Liposomes (phospholipids and SIM) in nanofibers represented 76% of the total formulation mass, thus significantly impacted the nanofiber morphology. Nanofibers with liposomes were thicker (more than 300 nm in diameter) with rougher surfaces, small sphere-like structures, and merged nanofiber cross-sections compared to homogenous EMPTY-NF with an average diameter of ~160 nm and a smooth surface. In addition, dried liposomes were visualized by TEM as bright spots homogeneously distributed over the nanofibers, an observation rarely reported in the scientific literature. Higher BHA content in the formulations significantly improved the chemical stability of SIM and accelerated SIM release compared to formulations with lower BHA content. After hydration of the nanofibers, cutting-edge MADLS^®^ analysis revealed two populations of liposomes, which is important since liposomes may enhance drug uptake into cells. In vitro viability assays showed that the nanofiber formulations were safer than the liposomal ones (both containing up to 4 µg/mL SIM) for both human keratinocytes and PBMCs. Additionally, both exerted comparable in vitro immunomodulatory activity on PHA-L-activated T lymphocytes, thereby showing potential for reducing tissue damage and promoting healing in inflamed chronic wounds. Altogether, our study provides insights into (i) the use of liposomes to obtain high SIM loading in alginate-based nanofibers, (ii) green electrospinning without organic solvents, (iii) liposome reconstitution after nanofiber hydration, and (iv) in vitro safety and efficacy of composite liposome–nanofiber formulations. Thus, the combination of nanofibers and liposomes led to the development of an innovative biocompatible nanodelivery system, which represents a promising and efficient dressing for chronic wound treatment.

## Figures and Tables

**Table 1:** Composition and characteristics of liposomes in terms of average diameter (nm), polydispersity index (PI), zeta potential (ZP, mV), and encapsulation efficiency (EE, %). Pairs of values of the same parameter that are significantly different are marked with the same small letter.

Formulations	Composition			Characterization			
	P90G (mg/mL)	SIM (mg/mL)	BHA (mg/mL)	Average Diameter (nm)	PI	ZP (mV)	EE (%)
SIM LIPO	100	20	0	65.1 ± 0.8 ^abc^	0.19 ± 0.01 ^ab^	−14 ± 3 ^a^	96 ± 5 ^ab^
0.3-BHA SIM LIPO	100	20	0.3	73.6 ± 4.0 ^de^	0.16 ± 0.01 ^acd^	−16 ± 1	88 ± 3
0.6-BHA SIM LIPO	100	20	0.6	77.9 ± 1.2 ^af^	0.16 ± 0.01 ^b^	−18 ± 1	88 ± 5
0.9-BHA SIM LIPO	100	20	0.9	86.4 ± 4.6 ^bdg^	0.18 ± 0.01 ^c^	−21 ± 2 ^a^	82 ± 5 ^a^
1.2-BHA SIM LIPO	100	20	1.2	105.9 ± 4.4 ^cefg^	0.17 ± 0.01 ^d^	−18 ± 3	80 ± 4 ^b^

**Table 2:** Characterization of the nanofibrous samples in terms of the average nanofiber diameter (nm), drug loading (DL, % *w*/*w*), and drug loading efficiency (DLE, %). Small letters indicate couples of statistically different values.

Formulations	Nanofiber Diameter (nm)	DL (% (*w*/*w*))	DLE (%)
EMPTY-NF	159.8 ± 25.2	-	-
SIM LIPO-NF	273.3 ± 64.6 ^ab^	10.61 ± 0.28	83.6 ± 2.4
0.6-BHA SIM LIPO-NF	315.4 ±79.0 ^a^	10.38 ± 0.45	80.9 ± 3.5
1.2-BHA SIM LIPO-NF	311.6 ±88.5 ^b^	10.41 ± 0.27	81.3 ± 2.0

**Table 3:** MADLS and particle concentration results of the dissolved LIPO-NF samples.

Formulations	Peak 1			Peak 2		
	Size (nm)	Intensity (%)	Particle Concentration (Particle/mL)	Size (nm)	Intensity (%)	Particle Concentration (Particle/mL)
SIM LIPO-NF	135.6	44.5	1.24 × 10^12^	432.2	39.0	2.46 × 10^10^
0.6-BHA-LIPO-NF	154.2	44.6	3.17 × 10^10^	438.0	45.7	1.31 × 10^11^
1.2-BHA-LIPO-NF	141.2	33.2	1.11 × 10^9^	436.3	48.8	2.12 × 10^12^

**Table 4:** Similarity factor (*f*_2_) of the SIM release profiles from the three formulations under sink conditions.

	0-BHA SIM LIPO-NF vs 0.6-BHA SIM LIPO-NF	0-BHA SIM LIPO-NF vs 1.2-BHA SIM LIPO-NF	0.6-BHA SIM LIPO-NF vs 1.2-BHA SIM LIPO-NF
*f*_2_	50	19	24

**Table 5:** Half-maximal inhibitory concentration (IC50) values of formulations tested on keratinocytes.

Formulation	IC50 (μg/mL)
EMPTY-LIPO	~1998
SIM LIPO	~47
1.2- BHA SIM LIPO	~9
SIM LIPO-NF	~323
1.2- BHA SIM LIPO-NF	~392

## Data Availability

Data available on request.

## Supplementary Materials

The following supporting information can be downloaded at: https://www.mdpi.com/article/10.3390/pharmaceutics15041245/s1, Table S1: Composition and characteristics of liposomes with different concentrations of P90G and SIM, in terms of average diameter (nm), polydispersity index (PI), and encapsulation efficiency (EE, %).
