# Supplementary material for: Development of Nanofibers with Embedded Liposomes Containing an Immunomodulatory Drug Using Green Electrospinning
Source: Pharmaceutics. 2023 Apr 14;15(4):1245. doi: 10.3390/pharmaceutics15041245 (PMC10143873; doi:10.3390/pharmaceutics15041245)
Supplement: Supplementary file 1 [file pharmaceutics-15-01245-s001.zip › pharmaceutics-2325575-supplementary.pdf]

pharmaceutics

MDPI

Supplementary Material

Development of Nanofibers with Embedded Liposomes Containing an Immunomodulatory Drug Using Green Electrospinning

Luca Casula [1,2], Anže Zidar [2], Julijana Kristl [2], Matjaž Jeras [2], Slavko Kralj [2,3,4], Anna Maria Fadda [1] and Špela Zupančič [2,*]

1. Unit of Drug Sciences, Department of Life and Environmental Sciences, University of Cagliari, Via Ospedale 72, 09124 Cagliari, Italy; luca.casula@unica.it (L.C.); mfadda@unica.it (A.M.F.)
2. Faculty of Pharmacy, University of Ljubljana, Aškerčeva Cesta 7, 1000 Ljubljana, Slovenia; anze.zidar@ffa.uni-lj.si (A.Z.); julijana.kristl@ffa.uni-lj.si (J.K.); matjaz.jeras@ffa.uni-lj.si (M.J.); slavko.kralj@ijs.si (S.K.)
3. Department for Materials Synthesis, Jožef Stefan Institute, Jamova Cesta 39, 1000 Ljubljana, Slovenia
4. Nanos SCI, Nanos Scientificae d.o.o., Teslova Ulica 30, 1000 Ljubljana, Slovenia

* Correspondence: spela.zupancic@ffa.uni-lj.si; Tel.: +386-1-4769500

Table S1. Composition and characteristics of liposomes with different concentrations of P90G and SIM, in terms of average diameter (nm), polydispersity index (PI), and encapsulation efficiency (EE, %). Samples indicated with "not measurable" showed high values of PI or macroscopic aggregates/precipitates and thus, were not suitable for DLS and/or EE% measurements.

FORMULATION OF P90G+SIM (both in mg/mL)	AVERAGE DIAMETER (nm)	PI	EE (%)
60 + 10	55.7 ± 3.7	0.19 ± 0.01	96 ± 12.7
60 + 15	326 ± 30.6	0.45 ± 0.07	57.1 ± 9
60 + 20	533.1 ± 72.4	0.95 ± 0.08	not measurable
80 + 10	50.4 ± 1.9	0.27 ± 0.01	100.8 ± 1.9
80 + 15	101.9 ± 26.6	0.26 ± 0.01	99.3 ± 1.9
80 + 20	473.6 ± 114.5	0.96 ± 0.1	not measurable
100 + 10	48.7 ± 1.8	0.33 ± 0.03	93.7 ± 3.6
100 + 15	47.5 ± 0	0.28 ± 0.01	95.7 ± 2.4
100 + 20	65.1 ± 0.8	0.19 ± 0.01	96.1 ± 5.1
100 + 25	not measurable		
120 + 20	129.9 ± 18.2	0.29 ± 0.1	92 ± 2.1
120 + 25	not measurable		
120 + 30	not measurable		
